# Haemophilia A: health and economic burden of a rare disease in Portugal

**DOI:** 10.1186/s13023-019-1175-5

**Published:** 2019-09-04

**Authors:** Andreia Café, Manuela Carvalho, Miguel Crato, Miguel Faria, Paula Kjollerstrom, Cristina Oliveira, Patrícia R. Pinto, Ramón Salvado, Alexandra Aires dos Santos, Catarina Silva

**Affiliations:** 1 Market Access & External Affairs, Roche Farmacêutica Química, Lda, Estrada Nacional 249 – 1, 2720-413 Amadora, Portugal; 2 Centro de Referência de Coagulopatias Congénitas do Centro Hospitalar Universitário S. João, EPE, Porto, Portugal; 3 Associação Portuguesa de Hemofilia e de Outras Coagulopatias Congénitas, Lisbon, Portugal; 4 Health Economics, Eurotrials Scientific Consultants, now part of CTI Clinical Trial & Consulting Services, Lisbon, Portugal; 5 Unidade de Hematologia Pediátrica, Hospital Dona Estefânia, Centro Hospitalar Lisboa Central, Lisbon, Portugal; 6 Centro de Coagulopatias Congénitas do Hospital de Santa Maria, Lisbon, Portugal; 7 Life and Health Sciences Research Institute (ICVS), School of Medicine, University of Minho, Braga, Portugal; 8 ICVS / 3B’s – PT GovernmentAssociateLaboratory, Braga / Guimarães, Portugal; 9 Centro de Coagulopatias Congénitas do Centro Hospitalar e Universitário de Coimbra (CHUC), Coimbra, Portugal; 10 Centro de Referência de Coagulopatias Congénitas do Centro Hospitalar Lisboa Central – Hospital São José, Lisbon, Portugal; 11 CISP - Centro de Investigação em Saúde Pública, ENSP – Universidade Nova de Lisboa, Lisbon, Portugal

**Keywords:** Haemophilia A, Cost-of-illness, Burden, Disability-adjusted life year, Portugal, Health economics

## Abstract

**Background:**

Haemophilia A is a hereditary bleeding disorder, which has been considered rare and chronic. The burden of this disease in Portugal remains unknown. The aim of this study was to estimate the annualized cost and health burden of haemophilia A in Portugal.

**Methods:**

Data were extracted from a Portuguese expert panel, from official data and national literature. Annual costs were calculated from the perspective of the society including direct and indirect costs. Unitary costs were extracted from 2017 national official sources and are expressed in euros. Health burden was expressed in disability adjusted life years (DALYs) based on incidence and quality of life questionnaires. Estimates are presented for the overall population and stratified by severity, age group (< 18 years vs. adults) and inhibitor status.

**Results:**

The yearly average cost per patient is estimated to range from €39,654/patient without inhibitors and €302,189/patient with inhibitors, representing a 7.6 fold difference. Amongst patients without inhibitors, the annual average cost was €401 in mild, €5327 in moderate and €85,805 in severe disease. Average cost per child and adult is €72,287 and €51,737, respectively. Direct costs represent approximately 95% of all costs, of which almost the totality accounts for clotting factor replacement therapy and bypassing agents. The total annual cost of haemophilia A for the Portuguese society was estimated to be €42,66 million, one third of which was related to the treatment of patients with inhibitors. It is estimated that haemophilia A is responsible for 3878 DALYs in Portugal (497 DALYs in mild, 524 DALYs in moderate, 2031 DALYs in severe patients without inhibitors and 784 DALYs in patients with inhibitors) for the cohort of 2017 (750 patients) or 5.2 DALY/patient during lifetime.

**Conclusions:**

Despite being rare, the economic and health burden of haemophilia A is remarkable. The main cost driver is clotting factor replacement therapy. Moreover, haemophilia A is more costly in children than in adults and rises exponentially with disease severity.

**Electronic supplementary material:**

The online version of this article (10.1186/s13023-019-1175-5) contains supplementary material, which is available to authorized users.

## Background

Haemophilia A is the most common severe inherited bleeding disorder characterized by spontaneous or traumatic bleeding owing to an inability to produce the clotting factor VIII. It is a rare genetic disease with an X-linked recessive pattern and thus affects primarily males (one in 5000–10,000 male births [1]) while females are carriers. Haemophilia is essentially an inherited bleeding disorder but one third of patients result from a de novo gene mutation [1]. Haemophilia A is traditionally classified as mild (6–40 IU/dL), moderate (1–5 IU/dL), or severe (< 1 IU/dL) [2, 3].

Bleeding episodes are the hallmark of haemophilia A and their extent depends on the severity of the disease and the presence/absence of neutralizing anti-factor VIII antibodies, known as inhibitors. Recurrent muscular bleeding and chronic joint disease is common in patients treated on demand, primarily as a result of bleeding into the joints, which can lead to long-term haemophilic arthropathies [4, 5]. These complications will lead to severe loss of function due to loss of motion, pain, deformity and activity limitation [4], affecting patients’ quality of life, psychological well-being and thus productivity [6, 7]. Orthopaedic complications usually require surgical interventions, such as orthopaedic surgery (total joint replacement), arthroplasty and mechanical or chemical synovectomy (synoviorthesis).

The standard treatment of haemophilia A is based on clotting factor replacement therapy on-demand (treatment for a bleeding episode control) or prophylaxis (continuous long-term treatment to prevent bleeding). Prophylactic treatment can lead to better clinical outcomes with better health-related quality of life and is changing the prognosis scenario, leading to a life expectancy close-to-normal in high- and middle- income countries [8, 9]. However, prophylactic treatment is more costly than on-demand therapy [10].

About 30% of patients with severe haemophilia A who are exposed to factor VIII concentrates will develop neutralizing anti-factor VIII antibodies (inhibitors), making clotting factor replacement therapy ineffective [11]. Development of inhibitors is the most serious complication of haemophilia and results in poor bleeding control and infeasible standard prophylaxis [12, 13], which subsequently means higher levels of morbidity and mortality and reduced health-related quality of life [10, 14, 15]. Inhibitor development risk is higher in patients with severe haemophilia A than in mild/moderate disease and mainly occurs in children [12]. Inhibitors have a great impact on therapeutic strategy, since patients with inhibitors require treatment with bypassing agents, namely recombinant activated factor VII (rFVIIa) or activated prothrombin complex concentrate (aPCC) [11]. Although both agents are considered effective, the efficacy of bypassing agents is suboptimal as compared with FVIII replacement therapy [12] and both options involve frequent intravenous infusions that depend on adequate venous access. However, the use of bypassing agents results in a significant increase to overall treatment costs [16, 17]. Immune tolerance induction (ITI) is the only strategy to eradicate high titre inhibitors (≥5 Bethesda units) and consists in the use of high doses of the clotting factor that triggered the development of inhibitors [17, 18].

Although other studies have estimated the health and/or economic burden of haemophilia A in other countries [14, 16, 18–23], differences in population, available resources, clinical practice and unitary costs vary considerably among countries, making this an important topic for nations to address individually.

To our knowledge, health and economic burden of haemophilia in Portugal have not yet been assessed. There was a Portuguese retrospective hospital-based study conducted using electronic patient records, but its aim was estimating only medical costs [13]. Considering the current economic setting in Portugal, the growing concerns about National Health Service sustainability [24], and the arrival of new treatment options for patients with haemophilia A, it is crucial to understand the current burden of this disease in Portugal, to help support priority setting in healthcare policies and resource allocation.

This study was conducted to pursue the following main objectives: (a) assess the economic burden of haemophilia A in Portugal from a societal perspective and (b) estimate the health burden of this disease by calculating the disability-adjusted life years (DALYs) or healthy life years lost, as a result of haemophilia A.

## Methods

### Data collection

Estimates for epidemiology data, resource use and labour absenteeism from informal caregivers were captured by an expert Delbecq panel [25] conducted in September 2017. Six experts from the most populated regions of Portugal (North, Centre and Lisbon and Vale do Tejo) with a large experience in treating haemophilia A patients were assembled.

Estimates were provided by age group (paediatric population with less than 18 years old and adults) whenever relevant. Patients were also stratified by presence (> 5 Bethesda Units) or absence of inhibitors. Patients without inhibitors were categorized by disease severity as mild (5–40%), moderate (1–5%), and severe (< 1%). In this analysis, bleeding episodes were categorized as *minor* (event not requiring hospitalization) or *major* (event requiring hospitalization).

To calculate treatment doses, mean body weight provided by the panel was used for adults. For children, weight was also provided by the panel considering pre-defined age groups (0 to 6 years-old, 7 to 12 years-old and 13 to 17 years-old). The distribution of children in each age group was obtained from national tables of male resident population from 0 to 17 years old for the year of 2017 [26].

Bleeding incidence and productivity loss in adult patients were captured using a cross-sectional survey conducted in Portugal with a sample of haemophilia A Portuguese patients. Surveys were answered between November 2016 and May 2017 and aimed to describe sociodemographic, clinical, and psychosocial characteristics of patients with haemophilia (PWH) of all ages in Portugal. One hundred and forty-six males with haemophilia A or B answered the survey: 106 adults, 21 children between 10 and 17 years, 11 children between 6 and 9 years and 8 children between 1 and 5 years. Further details about the survey are provided elsewhere [27].

### Cost analysis

Direct and indirect costs were estimated based on resource use resulting directly from haemophilia A and were characterized by the panel and literature [27].

Medical and non-medical direct costs were included and categorized as follows:

- Patient’s monitoring: consultations with health professionals (physicians, nurses, physiotherapists, etc.), laboratory and imaging exams, concomitant medications (for pain) and transportation to medical appointments;
- Prophylactic treatment: clotting factor replacement therapy;
- Bleedings: hospitalization (*major* bleedings), emergency room visits, follow-up visits and clotting factor replacement therapy;
- Orthopaedic complications: hospitalizations for surgical procedures (arthrodesis, arthroplasty and synovectomy);
- HIV co-infection related with haemophilia A.

Indirect costs were related exclusively with productivity loss due to haemophilia A and were calculated as follows:

- Unemployment rate: calculated within unemployed adult patients due to haemophilia A considering a productivity loss of 230 working days per year;
- Labour absenteeism: calculated within employed adult patients and informal caregiver considering the number of missing work days due to consultations and hospitalizations related with haemophilia A;
- Early retirement: calculated within early retired patients (less than 66 years old in Portugal) due to haemophilia A considering a productivity loss of 230 working days per year.

Employment and early retirement rates and absenteeism by patient per year in adult patients were obtained from the cross-sectional survey [27]. The employment rate exclusively for mild adult patients was obtained from Portuguese official sources for the general population [28] due to very low number of responses in the survey. Unemployment rate estimates for adult patients was not available from the survey, so these were provided by the experts. In children, only labour absenteeism from the informal caregiver was collected, which was provided by the experts due to very low number of responses in the survey. Employment rate for informal caregivers was also obtained from official national data [28]. Informal caregiver was defined in this study as the parent and/or responsible person for the child [27].

Unit costs were extracted from Portuguese official/public sources and are available in Additional file 1: Table S1.

Prices of drugs for hospital use were obtained from Catálogo Eletrónico de Aprovisionamento Público do SPMS [29] whereas prices of outpatient medication were extracted from INFOMED database [30] considering PVP/reference price and the most suitable pack size according to treatment duration. Price of clotting factors per UI was weighted by IMS market share in 2017. Costs for hospitalizations, consultations, labs and exams, physiotherapy sessions and home support were taken from the Portuguese DRG tables [31]. Transportation cost was calculated assuming a 100 km round trip [27, 32–34].

The cost of on-demand, prophylaxis and immune tolerance induction (ITI) treatment was estimated by multiplying the price per UI by patients’ weight and duration of treatment. The annual medical cost related with HIV was obtained from the literature [35, 36].

Indirect costs were valued based on the human capital approach using Portuguese wage tables from the National Institute of Statistics [37] by gender, considering the mean age of adult patients reported by the expert panel. Data on monthly net wages in the private sector, grossed up by employee’s and employers’ Social Security contributions and income taxes [38] was used to estimate 2017 monthly labour costs for employers. This was then multiplied by 14, to include the Portuguese standard vacation and Christmas extra monthly pay and divided by 223 annual working days (excluding weekends and official holidays). This cost was also considered for unemployed population. In paediatric population, work-related absences were attributed to informal caregivers. All costs are expressed in euros for the year of 2017.

### Calculation of DALYs

DALY represents the number of healthy life years lost due to haemophilia A and was estimated by the sum of two components: (a) number of years of life lost due to premature mortality (YLL) and (b) adjusted number of years lived with disability (YLD) associated with nonfatal haemophilia A [39]. DALY was computed using the prevalence-based approach employed in the 2010 Global Burden of Disease (GBD) study (DALY = YLL + YLD) considering the male population [39] and that patients are born with haemophilia A.

YLLs correspond to the number of deaths directly attributable to haemophilia A per year multiplied by the standard life expectancy at the age of death. The number of deaths per year was calculated based on expert opinion on the increased risk of death compared to Portuguese mortality rate for the year of 2016 [40]. Life expectancy at age of death was obtained using the West level 26 standard life table for men.

YLDs were estimated based on the number of incident cases of haemophilia A multiplied by disease duration or life expectancy multiplied by the severity of haemophilia A, disability weight (DW), on a scale from 0 (perfect health) to 1 (death).

DWs for health state quantify the severity of disease [41] and are dependent on social preferences. Several sets of DWs have been developed, such as the GBD DWs and the Dutch severity Weights [42]. Nonetheless, no haemophilia-specific DWs were available either in the Global Burden of Disease list, nor the Dutch Disability Weights Group.

The DW estimation was based on the results of the SF-6D scores generated from the SF-36 quality-of-life questionnaire filled out by 71 PWHs in Belgium [43] minus the mean SF-6D score for males in the general population (population norms) [44] and for each age groups. DWs were: 0.054, 0.151, 0.197 for mild, moderate or severe patients without inhibitors respectively and 0.500 for patients with inhibitors.

All calculations were made using the software provided by WHO [45]. A discount rate of 3% and weighting by age were applied. The method for estimating DALYs used for the Portuguese population follows the method proposed by WHO’s Global Burden of Diseases (GBD). Details about the data used for calculation may be found in Additional file 1: Table S10.

## Results

### Epidemiology

Overall, the experts (who were treating 674 haemophilia A patients at the time of the panel), estimated a total of 700 to 800 haemophilia A patients in Portugal. According to the experts, there are 10 new cases each year resulting in an incidence of haemophilia A in Portugal of approximately 22 per 100,000 male births [46]. Experts referred that life expectancy of patients with haemophilia A remains below that of the general male Portuguese population (75 years versus 78.1 years [47]). Nevertheless, this number is steadily increasing with the development of new treatments. The loss of 3.1 years of life per patient is mainly related to *major* bleedings, but also to HIV infection and HCV-related liver disease. Experts have the perception that patients treated on-demand have a considerably higher risk of death than patients on prophylactic treatment and that the former have a slightly higher death risk than the general Portuguese population, but were unable to quantify risks.

### Patients and disease characteristics

According to the experts, 25% of haemophilia A patients are children and 75% are adults. Distribution by severity is as follows: 37.5% mild patients without inhibitors, 14.2% moderate without inhibitors, 41.9% severe without inhibitors and 6.5% with inhibitors.

Children have a slightly higher proportion of severe cases (45.8% vs 40.6%) and inhibitors (8.4% vs 5.8%), compared to adults. Mean weight is 38.3 kg in children and 74 kg in adults. Adult patients are on average 43 years old. Additional information is detailed in Additional file 1: Table S2.

In the adult population, about 8 and 19% of patients are co-infected with HIV and HCV, respectively. These prevalence estimates are higher than in the general Portuguese population. The panel acknowledged that these co-infections were acquired in the past and the current incidence is null. Co-infections are confined to the group of patients with severe disease (with or without inhibitors).

### Clinical practice

#### Monitoring

Resource use in patients’ monitoring and disease management are described in Additional file 1: Table S3.

#### Treatment regimens

Therapeutic regimen is chosen based on disease severity and patient’s age. Patients’ distribution by type of regimen is described in Additional file 1: Table S4. According to the expert panel, the choice of FVIII replacement product for prophylaxis treatment depends on patient’s age. Details about prophylactic treatment are described in Additional file 1: Table S5. Administration of clotting factors for on-demand patients in *minor* and *major* bleedings is described in Additional file 1: Table S6.

#### Inhibitors

According to the expert panel, about 3 patients/year will develop inhibitors (which corresponds to an incidence of 0.5% among the total population or 1.6% in paediatric population), almost exclusively in paediatric population.

#### ITI (Immunotolerance induction) and bypassing agents

According to the expert panel, all patients with inhibitors start ITI treatment, which is used trying to eradicate inhibitors and consists in the administration of factor VIII that triggered the development of inhibitors, 100UI/kg/day for an average of 21 months (range: 6–36 months).

Bypassing agents are used to control bleeds in patients with inhibitors and can be used in prophylactic or on-demand regimens depending on patient’s age group. The choice of agent depends on patient’s age but not on the treatment regimen (prophylaxis or on-demand). Details of bypassing agent treatment are available in Additional file 1: Table S7.

#### Bleedings

When a *minor* bleed occurs, 40% of patients require medical attention (emergency or additional medical visit) and 15% will require a follow-up medical visit. Managing *major* bleeds requires hospitalization and 2–3 follow-up consultations. In muscle hematomas, patients undergo physiotherapy 3 times/week in the long-term. Details of bleeding by age group and treatment regimen are available in Additional file 1: Table S8.

#### Orthopaedic surgeries

The expert panel indicated that a large proportion of adults with severe disease with/without inhibitors have synovitis (80%) and chronic arthropathy (70%). These complications are not common in children (10 and 4%, respectively). About 12.5% of adults with severe disease without inhibitors are submitted to surgery each year. The number of surgeries per patient per year is on average 1–2. Patients with inhibitors are not usually submitted to surgeries, due to generally unfavourable risk-benefit.

### Productivity loss

Productivity loss estimates are provided in detail in Additional file 1: Table S9.

### Annual cost per patient

Annual cost per patient is estimated to be €56,875 of which €53,948 (94.9%) correspond to direct costs and €2927 (5.1%) to indirect costs. The latter are related with productivity loss of adult patients and informal caregiver, and were distributed as follows: 2% due to lost working days, 3% to unemployment and < 0.5% to early retirement.

Cost estimates by age group consists of €72,287 per paediatric patient and €51,737 per adult. In patients without inhibitors, yearly cost estimates are €401 in mild, €5327 in moderate and €85,805 in severe patients. The mean yearly cost per patient without inhibitors is €39,654 and €302,189 per patient with inhibitors. Cost results are described in Table 1.

**Table 1:** Annualized cost per patient

	Mild w/o inhibitors	Moderate w/o inhibitors	Severe w/o inhibitors	Inhibitors	Overall
Children					
Direct costs	€ 168	€ 295	€ 90,446	€ 349,973	€ 70,908
Monitoring	€ 168	€ 295	€ 724	€ 4,968	€ 835
Prophylaxis	€ 0	€ 0	€ 82,920	€ 40,478	€ 41,377
ITI	€ 0	€ 0	€ 0	€ 257,972	€ 21,670
Minor bleedings	€ 0	€ 0	€ 4,649	€ 38,469	€ 5,361
Major bleedings	€ 0	€ 0	€ 243	€ 8,086	€ 791
Orthopaedic surgeries	€ 0	€ 0	€ 1,910	€ 0	€ 875
HIV co-infection	€ 0	€ 0	€ 0	€ 0	€ 0
Indirect costs^a^	€ 0	€ 376	€ 2,347	€ 3,286	€ 1,379
Unemployment	€ 0	€ 0	€ 0	€ 0	€ 0
Early retirement	€ 0	€ 0	€ 0	€ 0	€ 0
Work absence	€ 0	€ 376	€ 2,347	€ 3,286	€ 1,379
Total cost	€ 168	€ 670	€ 92,793	€ 35, 260	€ 72,287
Adults					
Direct costs	€ 168	€ 5,661	€ 76,592	€ 279,429	€ 48,294
Monitoring	€ 168	€ 239	€ 644	€ 661	€ 402
Prophylaxis	€ 0	€ 5,422	€ 43,380	€ 48,576	€ 21,319
ITI	€ 0	€ 0	€ 0	€ 0	€ 0
Minor bleedings	€ 0	€ 0	€ 25,842	€ 224,212	€ 23,496
Major bleedings	€ 0	€ 0	€ 3,759	€ 4,923	€ 1,812
Orthopaedic surgeries	€ 0	€ 0	€ 1,910	€ 0	€ 775
HIV co-infection	€ 0	€ 0	€ 1,056	€ 1,056	€ 490
HCV co-infection	€ 0	€ 0	€ 0	€ 0	€ 0
Indirect costs	€ 310	€ 1,218	€ 6,884	€ 5,736	€ 3,443
Unemployment	€ 0	€ 51	€ 5,164	€ 5,164	€ 2,405
Early retirement	€ 0	€ 20	€ 30	€ 118	€ 22
Work absence	€ 310	€ 1,148	€ 1,690	€ 454	€ 1,016
Total cost	€ 478	€ 6,880	€ 83,476	€ 285,165	€ 51,737
Overall					
Direct costs	€ 168	€ 4,320	€ 80,055	€ 297,065	€ 53,948
Monitoring	€ 168	€ 253	€ 664	€ 1,738	€ 510
Prophylaxis	€ 0	€ 4,067	€ 53,265	€ 46,551	€ 26,333
ITI	€ 0	€ 0	€ 0	€ 64,493	€ 5,417
Minor bleedings	€ 0	€ 0	€ 20,544	€ 177,777	€ 18,962
Major bleedings	€ 0	€ 0	€ 2,880	€ 5,714	€ 1,557
Orthopaedic surgeries	€ 0	€ 0	€ 1,910	€ 0	€ 800
HIV co-infection	€ 0	€ 0	€ 792	€ 792	€ 368
Indirect costs	€ 233	€ 1,008	€ 5,750	€ 5,124	€ 2,927
Unemployment	€ 0	€ 38	€ 3,873	€ 3,873	€ 1,803
Early retirement	€ 0	€ 15	€ 23	€ 89	€ 17
Work absence	€ 233	€ 955	€ 1,854	€ 1,162	€ 1,107
Total cost	€ 401	€ ,5327	€ 85,805	€ 302,189	€ 56,875

^a^Informal caregivers

### Yearly economic burden

The overall expenditure of treating all haemophilia A patients in Portugal is estimated to be €42,655,976 of which 32% is attributable to paediatric population and 68% to adults (Table 2).

**Table 2:** Economic burden of haemophilia A in Portugal

	Mild w/o inhibitors	Moderate w/o inhibitors	Severe w/o inhibitors	Inhibitors	Overall
Children					
Direct cost	€12,121	€4,087	€7,767,020	€5,512,078	€13,295,306
Indirect cost	€0	€5,211	€201,579	€51,759	€258,549
Total	€12,121	€9,298	€7,968,599	€5,563,837	€13,553,855
Adults					
Direct cost	€35,185	€522,259	€17,491,655	€9,116,376	€27,165,476
Indirect cost	€64,913	€112,402	€1,572,189	€187,141	€1,936,645
Total	€100,099	€634,661	€19,063,844	€9,303,517	€29,102,121
Overall					
Direct cost	€47,306	€526,346	€25,258,675	€14,628,455	€40,460,782
Indirect cost	€64,913	€117,613	€1,773,768	€238,900	€2,195,194
Total	€112,219	€643,959	€27,032,443	€14,867,354	€42,655,976

About 0.3% of haemophilia A expenditure is associated to mild cases without inhibitors (37.5% of all patients), 1.5% to moderate cases without inhibitors (14.2% of all patients), 63.4% to severe cases without inhibitors (41.9% of all patients) and 34.9% to patients with inhibitors (6.5% of all patients).

Prophylaxis and on-demand regimens represent 46 and 35% of the total yearly haemophilia A cost, respectively.

### Disability-adjusted life years (DALY)

This study estimated that a total of 3878 DALYs in males are attributable to haemophilia A for the cohort of 2017 (or 5,2 DALY/patient during lifetime). Of these, 3835 (99%) were YLD and the remaining 43 (1%) were YLL. Patients with severe haemophilia A account for more than 52% of DALYs, whereas patients with inhibitors account for 20%. Haemophilia A health burden is described in Table 3.

**Table 3:** Health burden of haemophilia A in Portugal

	Base-case	Sensitivity analysis		
	3% time discounting, age weighted	0% time discounting, no age weighted	3% time discounting, no age weighted	0% time discounting, age weighted
Total YLLs	43	92	79	49
Total YLDs	3,835	8,789	3,494	9,725
Mild	497	1,138	452	1,259
Moderate	524	1,200	477	1,328
Severe	2,031	4,654	1,850	5,150
With inhibitors	784	1,796	714	1,987
Total DALYs	3,878	8,880	3,573	9,774

## Discussion

This study was performed using detailed data about epidemiology, resource consumption and productivity loss related with haemophilia A. Our findings confirm that management of this disease is costly, namely when patients develop inhibitors. The study suggests that haemophilia A imposes a significant economic burden on Portuguese NHS and subsequently, on society.

To our best knowledge this is the first study that attempts to characterize the health and economic burden of haemophilia A in Portugal approaching direct/indirect costs and DALYs.

We have chosen the broader societal perspective, which includes costs supported by the NHS and Social Security for patients/families. We found an annualised mean cost of €56,875 per patient with haemophilia A in Portugal in the societal perspective and €53,948 in the perspective of the NHS. This estimate is about 34 times higher than the public health expenditure per capita in Portugal of approximately €1602/year/person [48].

Direct comparison with studies performed in other countries is limited, as mentioned in the Background section, but it is however possible to analyse trends in costs. Considering the proportion between direct costs and indirect costs estimated in our study (95% vs. 5%) similar results had already been reported in previous studies [19, 20]. This is not surprising, since (a) the disease does not greatly interfere with professional activity in mild/moderate cases (approximately half of the population with haemophilia A) and (b) productivity loss has a low value compared with treatment costs due to high intensity of treatment, especially in prophylactic regimens, and cost of drugs. Our estimations show that prophylactic procedures, ITI treatment and *minor* bleeding treatment are the *major* cost drivers, accounting for 89% (€50,712) of the overall annual cost/patient. This finding is in accordance with results found by O’Hara [18], Henrard [19] and Rocha [13] et al., the latter of which estimated an average cost of €44,134 per patient-year in a Portuguese hospital setting. Considering direct costs alone, these treatment resources account for nearly 94% of costs, a proportion similar to the previously described for Spain, Germany, Hungary and the UK by Cavazza et al. [22]

Considering a total of 750 haemophilia A patients in Portugal and the annual cost obtained per patient, it is expected that around 40.4 million euros are spent every year by the NHS in the treatment of this disease. This amount corresponds to around 0.4% of the national health budget estimated for 2017, which consisted of 9.9 million € with additional 2.2 million euros from lost productivity [49]. Assuming that the true haemophilia population ranges from a minimum of 539 [50] to a maximum of 800 patients as reported by the experts, the true economic burden of haemophilia A to society may range between €31.8 million to €45.2 million. The high impact of the population with inhibitors to the economic burden of haemophilia A should be highlighted, since about 35% of haemophilia A societal annual expenditure is related with these patients, who represent only 6.5% of haemophilia A population.

Comparing different age groups, the annual cost per patient is 39% higher in children than adults. This can be explained by: (a) development of inhibitors mainly occurring in childhood; (b) consequent ITI administration and; (c) children being mostly treated with rFVIIa which is a more expensive treatment than aPCC, while the majority of adults are treated with aPCC. Nerich et al. [14] also found differences in costs between age cohorts, but this study only included severe cases. The author identified that children have a higher consumption of clotting factor/kg because, on average, they may experience more bleeding episodes than adults, with more extensive secondary disease prevention and a less favourable post-surgical recovery. In the study performed by Rocha et al. [13] in a Portuguese hospital, the total annual cost/adult (€49,338) was very similar to our estimate but the cost/paediatric patient (€37,805) was about half of our result with no statistically difference between adults and children (*p* = 0.47). Rocha provides three possible explanations for the similar costs obtained in adults and children: the vast majority of children were teenagers and their body weight would be comparable to adults, different pharmacokinetics of clotting factor in children leading to a high consumption of clotting factor/kg in children and prophylaxis was used in 100% of children/teenagers versus 40–70% in adults with severe disease.

Cost comparisons by severity showed that yearly costs increase exponentially with severity of disease ranging from €401 per mild patient to €302,189 per patient with inhibitors. Our annual direct cost estimates per mild, moderate and severe (+/− inhibitors) patients are different from those found by Rocha et al. [13] (€793, €8,495 and €77,587, respectively). Nevertheless, increments between severity levels follow a similar trend.

The development of inhibitors results in a great impact to the economic burden of the disease. Our data suggests that a patient with inhibitors costs 7.6 times more than a patient without inhibitors. This can be explained by: (a) higher incidence of bleeding episodes requiring consumption of clotting factor; (b) bypassing agents rFVIIa and aPCC for patients with inhibitors are more costly than plasma-derived FVIII and rFVIII for patients without inhibitors and; (c) need of ITI. Surprisingly, our estimate for the cost per patient with inhibitors was about the double of that described by Rocha et al. (€134,032) [13] but we obtained a similar cost per patient without inhibitors (€40,318).

Additionally, in our study, the cost of severe patients without inhibitors was 3.5 times lower than patients with inhibitors, which can also be explained by b) and c) above. Similarly, Nerich et al. [14] found that direct drugs costs in severe patients without inhibitors were two times lower than the cost of patients with inhibitors.

DALY is the primary metric used by WHO to assess the global burden of disease. The years lived with disability have the highest contribution to haemophilia-related DALYs (99%) whereas the years of life lost due to premature mortality were considerably low (1%). This result was expected, since life expectancy of patients with haemophilia A is close to that of general population due to great advances in the development of safer FVIII replacement products. The large contribute of YLDs in total DALYs demonstrate that patients with haemophilia A have poorer quality of life compared with general Portuguese population. In our study, patients with inhibitors had the largest DALY estimate per patient. Improving the quality-of-life and avoiding premature mortality is critical in these patients.

The results show that DALY estimation is largely dependent on the assumptions made in the calculation, namely, discount and weighting of age. DALYs are strongly related with the prevalence of diseases and therefore, DALYs from haemophilia A are normally lower since this is a rare disease. Further research and quantification of burden of disease in DALYs using disease specific disability weights, when available, will make the estimated burden more applicable to people with haemophilia A.

The strengths of this study are the comprehensive inclusion and description of all relevant components of cost when performing the cost-of-illness study, the use of real-world data to estimate bleeding frequency and the inclusion of productivity loss in adult patients and informal caregiver’s absenteeism. Given that many bleeding episodes are self-controlled by the patient, the use of patients’ self-reported frequency of bleeding was preferred compared to the information collected from the panel, since the latter was based on physician perception [27].

Nevertheless, there are some limitations of our study that should be pointed out.

This study was partially based on data provided by an expert panel. This is a valid method that is widely used in several fields including health sciences. Experts were invited to a face-to-face meeting to present individual data based on their own casuistic regarding management of haemophilia A. Discussion between experts was encouraged by an independent medical moderator, namely to understand differences found between population of patients or practices. Since experts were treating 90% of the entire haemophilia A population in Portugal, the data herein presented is considered consistent, representative and without selection bias.

The cross-sectional survey had some limitations, namely, sample size by severity group, a one-year recall period, potential selection bias due to voluntary participation and missing values. The survey showed a trend for an increase in productivity loss with the severity of the disease, except when we move from adults with severe disease to adults with inhibitors probably due to missing data in lost working days. Nevertheless, it should be stressed that this observational study included 16% of the Portuguese population with haemophilia A.

Several other resources were not covered by this study such as loss of earnings by other informal caregivers (e.g. partners, other relatives of adult patients), use of a professional caregiver, emergency transportation in case of bleedings, home changes, alternative therapies (such as massage, nutritionist or homeopathy), treatment of adverse events resulting from anti-haemophilic drugs, presenteeism, premature death or intangible costs related with the disease such as anxiety, pain, reduced quality of life and suffering of patient and family. Further studies are needed to estimate the costs mentioned above on a proper and robust manner.

Moreover, experts mentioned other haemophilia A complications in adults besides bleeding, such as foot ulcer or limb pseudotumor that may result in amputation prosthesis, wheelchairs use, technical help, and orthotics, which were not quantified in this study.

Costs related with HCV co-infections were not considered, since we assumed, as reported by the experts, that in 2017 there were no new cases and all co-infected were successfully treated in the past. Nevertheless, re-treatments may occur in HCV and therefore costs are probably underestimated but were not possible to be quantified by the experts. Regarding HIV infection, estimates used for the HIV annual treatment cost did not include indirect costs and we have assumed only haemophilia A productivity loss to avoid double counting.

We have assumed that patients are 100% compliant with treatments but this assumption may not be valid in the real-world, resulting in lower treatment costs.

We were not able to find data about unemployment of parents related with having children with haemophilia A.

Hospitalization, consultations and emergency costs correspond to hospital reimbursement costs and were used as a proxy of the real cost. Nevertheless, these costs are irrelevant compared with clotting factor. Prices used for clotting factor are also a limitation since hospital tenders may decrease prices.

Weight is an extremely important factor when calculating the cost of clotting factor. Since we have not found any national available source describing weight by year of age, it was not possible to have the desirable granularity over this parameter. The impact of using a constant weight within age intervals should be further investigated with sensitivity analysis.

Although we recognise several limitations in the estimation of the economic burden, given the proximity between our results and other reported in previous studies and considering the wide national representativeness of the experts that provided the data, we believe that estimates from our study are accurate and robust and may be considered as representative of the Portuguese population with haemophilia A.

For the DALY calculation, a limitation relies on the fact that disability weights are a crucial factor in YLD calculation as they allow for adjustment of the number of years lived with disability and for comparison with the number of life years lost due the premature mortality. Unfortunately, these weights are not available for Portugal and estimates may lack external validity due to country specificities.

## Conclusions

Despite being rare, haemophilia A remains one of the most costly and challenging diseases to manage. This study allowed to quantify costs and number of years lost due to morbidity or early death from haemophilia A for all patients and contributed to the understanding of differences between age groups, severity of disease and the presence or absence of inhibitors.

This study provides an important insight into the current clinical practice regarding management of haemophilia A in Portugal and highlights the wide range of resources that are needed to manage this rare disease. The larger contributor to the economic impact of the disease is the cost of treatment and indirect costs that were collected did not have a substantial impact on the results. However, considering the study limitations and the lack of data, further research with a more exhaustive collection of other indirect costs could lead to different conclusions.

We believe this study is relevant, since it is aligned with the growing interest of authorities in rare and chronic diseases such as haemophilia A, namely in what concerns national management strategies and may be useful in defining new policies and support prioritization on resource allocation decisions, considering also the upcoming availability of new treatments for these patients. Moreover, this study is an important contributor in encouraging and leveraging the conduction of real-world data studies on haemophilia A, namely, based on national registries.

## Additional file

Additional file 1: **Table S1.** Unitary cost per resource. **Table S2.** Patient characteristics**. Table S3.** Yearly health resource use in monitoring. **Table S4.** Patients’ distribution by treatment regimen and severity. **Table S5.** Treatment in prophylaxis. **Table S6.** Treatment of minor/major bleedings. **Table S7.** Treatment with bypassing agents. **Table S8.** Annualised bleeding rate and number of bleedings. **Table S9.** Estimates for calculation of indirect costs. **Table S10.** DALYs calculation. (DOCX 60 kb)

## Data Availability

Data sharing is not applicable to this article as no datasets were generated or analysed during the current study.

## Abbreviations

aPPC: Activated Prothrombin Complex Concentrate
DALYs: Disability-adjusted Life Years
DW: Disability Weight
GBD: Global Burden of Disease
ITI: Immune Tolerance Induction
NHS: National Health Service
rFVIIa: Recombinant Activated Factor VII
YLD: Years Lived With Disability
YLL: Years of Life Lost
