# Supplementary material for: Haemophilia A: health and economic burden of a rare disease in Portugal
Source: Orphanet J Rare Dis. 2019 Sep 4;14:211. doi: 10.1186/s13023-019-1175-5 (PMC6727364; doi:10.1186/s13023-019-1175-5)
Supplement: Supplementary file 1 — Table S1. Unitary cost per resource. Table S2. Patient characteristics. Table S3. Yearly health resource use in monitoring. Table S4. Patients’ distribution by treatment regimen and severity. Table S5. Treatment in prophylaxis. Table S6. Treatment of minor/major bleedings. Table S7. Treatment with bypassing agents. Table S8. Annualised bleeding rate and number of bleedings. Table S9. Estimates for calculation of indirect costs. Table S10. DALYs calculation. (DOCX 60 kb) [file 13023_2019_1175_MOESM1_ESM.docx]

**ADDITIONAL TABLES**

**Table S1 Unitary cost per resource**

| **Resource** | **Unitary cost** | **Source** |
| --- | --- | --- |
| Hospitalization due to intracranial bleeding | € 6,263.77 | (31) |
| Hospitalization due to other bleedings | € 3,003.10 | (31) |
| Hospitalization due to orthopaedic surgery | € 10,185.75 | (31) |
| Emergency room visit | € 112.07 | (31) |
| Specialist visit | € 31.00 | (31) |
| Other health care professional visit | € 16.00 | (31) |
| Phisiotherapysessions | € 22.40 | (31) |
| Investigation/titrationofinhibitors | € 113.90 | (31) |
| Factordosage | € 53.60 | (31) |
| Pharmacocinetics for prophylaxisindividualization | € 8.80 | (31) |
| Hepatitis A, B, C antibodies | € 61.85 | (31) |
| HIV 1 and 2 antibodies | € 18.10 | (31) |
| Othertests* | € 25.20 | (31) |
| Plasma-derived FVIII products (per UI) | € 0.3200 | (31) |
| Recombinant FVIII products (per UI) | € 0.5794 | (31) |
| rFVIIa (per µg) | € 0.5765 | (31) |
| aPCC (per UI) | € 0.7650 | (31) |
| Paracetamol (20 tablets) | € 1.79 | (31) |
| Tramadol (60 tablets) | € 6.36 | (31) |
| Celecoxib (60 tablets) | € 9.91 | (31) |
| Home assistance(per day) | € 33.10 | (31) |
| Travel cost/km | € 0.20-€ 0.40 | (46–48) |
| Working day, male | € 114.64 | (34,35) |
| Working day, both genders | € 104.10 | (34,35) |

*Prothrombin time, activated partial thromboplastin time, complete blood test, ALT, AST, gamma-GT, total cholesterol, iron, ferritin, transferrin saturation

**Table S2 Patient characteristics**

|  | **Children** | | | **Adults** | **Source** |
| --- | --- | --- | --- | --- | --- |
|  | **0 to 6 years old** | **7 to 12 years old** | **13 to 17 years old** |  |  |
| Mean weight | 19.0 kg | 36.0kg | 63.0kg | 74.0kg | Expert panel |
| Mean age | Not applicable* | Not applicable* | Not applicable* | 43 years | Expert panel |
| Total population (%) | 326,837 (35%) | 316,328 (34%) | 285,237 (31%) | Not applicable (assumed 100%) | (26) |

**Table S3 Yearly health resource use in monitoring**

|  | **Children** | | | | **Adults** | | | |
| --- | --- | --- | --- | --- | --- | --- | --- | --- |
| **Resources** | **Mild w/o inhibitors** | **Moderate**  **w/o inhibitors** | **Severe**  **w/o inhibitors** | **With inhibitors** | **Mild w/o inhibitors** | **Moderate**  **w/o inhibitors** | **Severe**  **w/o inhibitors** | **With inhibitors** |
| Specialist visit (coagulopathy) | 1 | 1 | 2.5 | 6 | 1 | 1 | 1.5 | 1.5 |
| Paediatrician visit | 0 | 0 | 0 | 0 | - | - | - | - |
| Orthopaedist visit | 0 | 0 | 1 | 2 | 0 | 0 | 1.5 | 2 |
| Physiatrist visit | 0 | 0 | 1.5 | 1.5 | 0 | 0 | 3.5 | 3.5 |
| Nurse visit | 0 | 0 | 2.5 | 6 | 0 | 0 | 1.5 | 1.5 |
| Dentist visit | 0 | 0 | 0 | 0 | 0 | 0 | 0 | 0 |
| Psychology visit | 0 | 0.2 | 0.3 | 0.3 | 0 | 0 | 0 | 0 |
| Investigation/titration of inhibitors | 0.5 | 1 | 2 | 12 | 0.5 | 1 | 2 | 2 |
| Factor dosage | 0.5 | 1 | 2 | 12 | 0.5 | 0 | 0 | 0 |
| Pharmacokinetics for prophylaxis individualization | 0 | 0 | 0.17 | 0 | 0 | 0 | 0 | 0 |
| Hepatitis A, B, C antibodies | 0 | 0.5 | 1 | 1 | 0 | 0.5 | 1 | 1 |
| HIV 1 and 2 antibodies | 0 | 0.5 | 1 | 1 | 0 | 0.5 | 1 | 1 |
| Other tests* | 1 | 1 | 1.5 | 1.5 | 1 | 1 | 1.5 | 1.5 |
| Physiotherapy sessions | 0 | 0 | 0 | 0 | 0 | 0 | 1.2 | 1.2 |
| Home assistance | 0 | 0 | 0 | 1 | 0 | 0 | 0 | 0 |
| Transportation | 1 | 1 | 2.5 | 6 | 1 | 1 | 1.5 | 1.5 |
| Pain relief medication** | 0 | 0 | 0 | 0 | 0 | 0.1 | 0.7 | 0.9 |

*Prothrombin time, activated partial thromboplastin time, complete blood test, ALT, AST, gamma-GT, total cholesterol, iron, ferritin, transferrin saturation

**Paracetamol, tramadol and celecoxib

**Table S4 Patients’ distribution by treatment regimen and severity**

| **Severity** | **Children** | | **Adults** | |
| --- | --- | --- | --- | --- |
|  | On demand | Prophylaxis | On demand | Prophylaxis |
| Mild without inhibitors | 100% | 0% | 100% | 0% |
| Moderate without inhibitors | 100% | 0% | 95% | 5% |
| Severe without inhibitors | 0% | 100% | 60% | 40% |
| With inhibitors | 90% | 10% | 90% | 10% |

**Table S5 Treatment in prophylaxis**

| **Age group** | **Severity** | **Treatment** | **Posology** |
| --- | --- | --- | --- |
| Children | Severe w/o inhibitors | 10% plasma-derived factor VIII | 20-30 UI/kg, 3x/week |
|  |  | 90% recombinant factor VIII | 20-30 UI/kg, 3x/week |
|  | With inhibitors | 90% rFVIIa | 90-120 µg/kg, eod |
|  |  | 10% aPCCs | 50-75 UI/kg, 2-3x/ week |
|  |  |  |  |
| Adults | Moderate w/o inhibitors | 50% plasma-derivedfactor VIII | 20-30 UI/kg, 2-3x/ week |
|  |  | 50% recombinant factor VIII | 20-30 UI/kg, 2-3x/ week |
|  | Severe w/o inhibitors | 50% plasma-derivedfactor VIII | 20-30 UI/kg, 2-3x/ week |
|  |  | 50% recombinant factor VIII | 20-30 UI/kg, 2-3x/ week |
|  | With inhibitors | 20% rFVIIa | 90-120 µg/kg, eod |
|  |  | 80% aPCCs | 50-75 UI/kg, 2-3x/week |

Abbreviations: eod - every other day

**Table S6 Treatment of minor/major bleedings**

|  | **Regimen** | **Factor used** | **Dosage** |
| --- | --- | --- | --- |
| **Minor bleeding** |  |  |  |
| Without inhibitors | On-demand | Previous untreated patients: Recombinant FVIII products or Plasma-derived FVIII products or analogues of vasopressin or antifibrinolytics;  Previously treated patients: usual treatment (used previously). | 20-30IU/Kg (1 to 3) |
|  | Prophylaxis | Therapy usually done in prophylaxis and/or antifibrinolytics | 20-30IU/Kg (1 extra dose) |
|  |  |  |  |
| With inhibitors | On-demand | Bypassing agent and/or antifibrinolytics agents: |  |
|  |  | rFVIIa | 90 µg/kg, 2/2h, 1-3 administration or 270 µg/kg single dose. |
|  |  | aPCC | 50-100 UI/kg (average: 75), 1-3 administrations  (should not exceed 200 IU/kg/day). |
|  | Prophylaxis | Bypassing agent and/or antifibrinolytics agents: |  |
|  |  | rFVIIa | 90 µg/kg 2/2h, 1-3 administration or 270 µg/kg single dose |
|  |  | aPCC | 50-100 UI/kg (average: 75), 1-3 administrations (should not exceed 200 IU/kg/day) |
| **Major bleeding** |  |  |  |
| Without inhibitors | Temporary prophylaxis | Therapy usually done in prophylaxis | 20-30 IU/kg - 1 to 6 weeks (3x/week or daily, higher than it did before). |
|  | Continuous prophylaxis | Common doses of prophylaxis |  |
|  |  |  |  |
| With inhibitors | Temporary prophylaxis | Bypassing agent and/or antifibrinolytics agents: |  |
|  |  | rFVIIa | 90-120 µg/kg every other day;1 to 6 weeks. |
|  |  | aPCC | 50-75 UI/kg; 1 to 6 weeks. |

**Table S7 Treatment with bypassing agents**

| **Treatment** | **Children** | **Adults** | **Regimen** | **Posology** |
| --- | --- | --- | --- | --- |
| rFVIIa | 90% | 20% | 90% on-demand | Outpatient: 90 µg/kg, 2/2h, 2-3 times or 270 µg/kg single dose;  Inpatient: if *major* bleeding |
|  |  |  | 10% in prophylaxis | 90-120 µg/kg every other day |
| aPCCs | 10% | 80% | 90% on-demand | 50-100 UI/kg, 1-3 times (maximum 200 UI/kg/day) |
|  |  |  | 10% in prophylaxis | 50-75 UI/kg, 2-3x/week |

**Table S8 Annualised bleeding rate and number of bleedings**

|  | **Children** | | | | **Adults** | | | |
| --- | --- | --- | --- | --- | --- | --- | --- | --- |
|  | **Severe w/o inhibitors** | | **With inhibitors** | | **Severe w/o inhibitors** | | **With inhibitors** | |
|  | % patients | No. | % patients | No. | % patients | No. | % patients | No. |
| **On-demand** |  |  |  |  |  |  |  |  |
| Minor bleeding | 75% | 5 | 100% | 8 | 94% | 22 | 100% | 26 |
| Major bleeding | 7% | 1 | 33% | 1 | 15% | 2 | 11% | 1 |
|  |  |  |  |  |  |  |  |  |
| **Prophylaxis** |  |  |  |  |  |  |  |  |
| Minor bleeding | 73% | 5 | 100% | 4 | 77% | 7 | 100% | 20 |
| Major bleeding | 6% | 1 | 10% | 1 | 12% | 2 | 0% | 0 |
|  |  |  |  |  |  |  |  |  |

**Table S9 Estimates for calculation of indirect costs**

|  | **Mild**  **w/o inhibitors** | **Moderate**  **w/o inhibitors** | **Severe**  **w/o inhibitors** | **With inhibitors** | **Souce** |
| --- | --- | --- | --- | --- | --- |
| **Adults**(27) |  |  |  |  |  |
| % of patients employed | 90.2%* | 77% | 67% | 36% | (27), (28) |
| Absenteeism by patients/year haemophilia A-related | 3 days | 13 days | 22 days | 11 days | (27) |
| Unemployment haemophilia A-related | 0% | 0% | 20% | 20% | Expert Panel |
| Early retirement rate haemophilia A-related | 0% | 7% | 14% | 55% | (27) |
|  |  |  |  |  |  |
| **Children (from informal caregivers)** |  |  |  |  |  |
| % of parents employed* | 90.2% | 90.2% | 90.2% | 90.2% | (28) |
| Absenteeism by informal caregivers/year | 0 days | 4 days | 25 days | 35 days | Expert Panel |

*Assumed employment rate of the Portuguese population(28).

**Table S10 DALYs calculation**

| **Variables** | **Values** | **Source** |
| --- | --- | --- |
| Mortality during the relevant year (number) | 9 | Expert opinion |
| Life expectancy (years) | 75 | Expert opinion |
| Life expectancy at age of death (years) | 10.17 | West level 26 standard life table for men |
| Age at onset | At birth (0) | Assumption since it is a genetic disease |
| Duration of disability s | 75 | Difference from Life expectancy and Age at onset |
| Mortality rate per 1,000 (2016) | 10,7 | (49) |
| **Disability weight** |  |  |
| Mild | 0.054 | (50) |
| Moderate | 0.151 | (50) |
| Severe | 0.197 | (50) |
| Severe with inhibitors | 0.500 | (50) |
| **DALY parameters** |  |  |
| Discount rate | 3% | WHO recommendation |
| Age weight | Yes | WHO recommendation |
| Constant | 0.1658 | WHO recommendation |
| -(b+r) | -0.07 | WHO recommendation |
| Beta(β) | 0.04 | WHO recommendation |
